# Validation of an electronic early warning score using decision tree analysis: proposal

**DOI:** 10.1186/cc14583

**Published:** 2015-03-16

**Authors:** M Xu, B Tam, L Thabane, AE Fox-Robichaud

**Affiliations:** 1 McMaster University, Hamilton, ON, Canada

## Introduction

**Figure 1:**
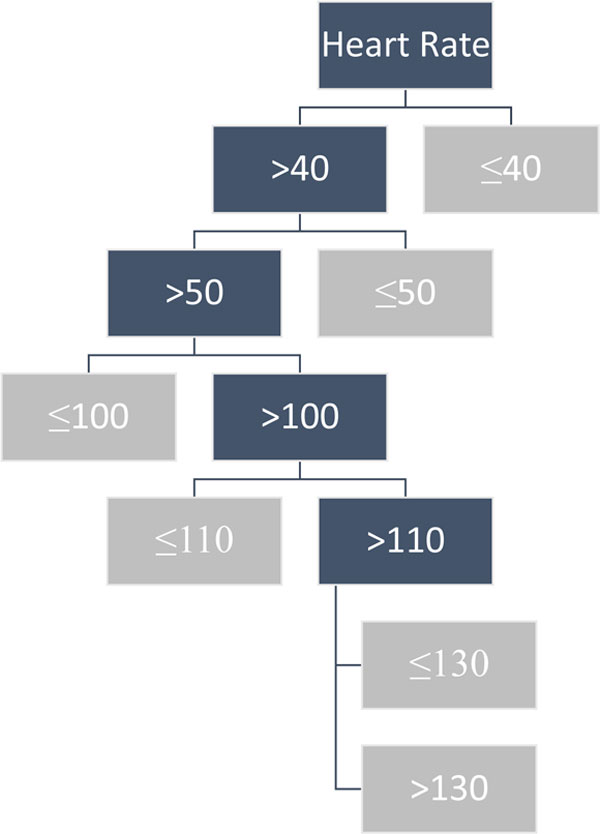
**Sample heart rate decision tree**.

Decision tree analysis uses an algorithm to classify data items by recursively posing a series of questions about items within a dataset. Each question leads to another node and potentially more questions until a predefined end condition is reached or no more questions can be asked (Figure 1). We hypothesize that scores generated using the decision tree method will improve upon our existing Hamilton Early Warning Score (HEWS) for a composite endpoint of cardiac arrest, unplanned ICU admission or death.

## Methods

A database of 156,642 electronically captured vital signs from 6,757 consecutively admitted patients to eight medical and surgical wards will be used to train and test the decision tree early warning score. One-third of the data will be withheld from the algorithm for use as a testing set. The algorithm will look for significant changes in vitals 72 hours prior to an outcome and develop the score based upon the resulting relative risk of the composite endpoint happening given a certain vital sign. The scores and predictions generated by the decision tree analysis will then be compared with that of the inception HEWS cohort.

## Results

The planned analysis for determining the discriminatory and predictive ability of the decision tree HEWS will be conducted with area under the receiver operating characteristic curves. We will test whether the current HEWS has the appropriate sensitivity and specificity when compared with that of the decision tree score. The AUROC will be calculated for both the training set of data as well as the separate population of additional medical and surgical patients. The two scores will also be plotted along an efficiency curve, comparing the percentage of vitals that precede a critical event with the percentage of vitals that produce a EWS value greater than or equal to a given EWS value.

## Conclusion

Decision tree analysis methodology with real-life vital signs can produce an EWS superior to previous observational studies. Using a decision tree, especially one that composites all vitals, may show that certain vitals are more predictive of critical events than others. Data will be used to further improve our current HEWS score.

